# Tetra­kis{2-[2-(2,6-dichloro­anilino)phen­yl]ethano­ato-κ^2^ *O*:*O*′}bis­[(dimethyl sulfoxide-κ*O*)copper(II)](*Cu*—*Cu*): a binuclear Cu^II^ complex with the non-steroidal anti-inflammatory drug diclofenac

**DOI:** 10.1107/S160053681201152X

**Published:** 2012-03-24

**Authors:** Stéphanie Sayen, Emmanuel Guillon

**Affiliations:** a Institut de Chimie Moléculaire de Reims, ICMR, UMR CNRS 7312, Université de Reims Champagne Ardenne, Moulin de la Housse, BP 1039, 51687 Reims cedex 2, France

## Abstract

The title compound, [Cu_2_(C_14_H_10_Cl_2_NO_2_)_4_(C_2_H_6_OS)_2_], comprises a Cu^II^_2_ core that is quadruply bridged by four carboxyl­ate ligands with the dimethyl sulfoxide ligands binding along the Cu⋯Cu axis. The four carboxyl­ate ligands bind in a bidentate *syn–syn* bridging mode. Mol­ecules reside on crystallographic inversion centres bis­ecting the mid-point of the Cu⋯Cu axis. There are no inter­molecular inter­actions of note.

## Related literature

Cu^II^ complexes of non-steroidal anti-inflammatory drugs (NSAIDs) show enhanced anti-inflammatory activity and reduced gastrointestinal toxicity compared with their uncomplexed parent drug, see: Weder *et al.* (2002 ▶). The structure of the Cu–NSAID is likely to be an important factor for its biological activity. For example, the anti-tumor activity of the monomeric Cu^II^ complex of aspirin ([Cu(Asp)_2_(py)_2_]) is reportedly more effective than the dimeric [Cu_2_(Asp)_4_] complex, see: Oberley & Buettner (1979 ▶). It has been shown that dinuclear Cu–NSAID complexes exhibit similar bio­logical activity to mononuclear complexes, but with higher stability (Dimiza *et al.*, 2011 ▶), making them relevant compounds in the treatment of tumor cell lines (Theodorou *et al.*, 1999 ▶). For mono- and binuclear Cu^II^ complexes of diclofenac, see: Sayen *et al.* (2012 ▶) for [Cu(diclofenac)_2_(H_2_O)_2_]·2H_2_O and Kovala-Demertzi *et al.* (1997 ▶) for [Cu_2_(diclofenac)_4_(DMF)_2_].
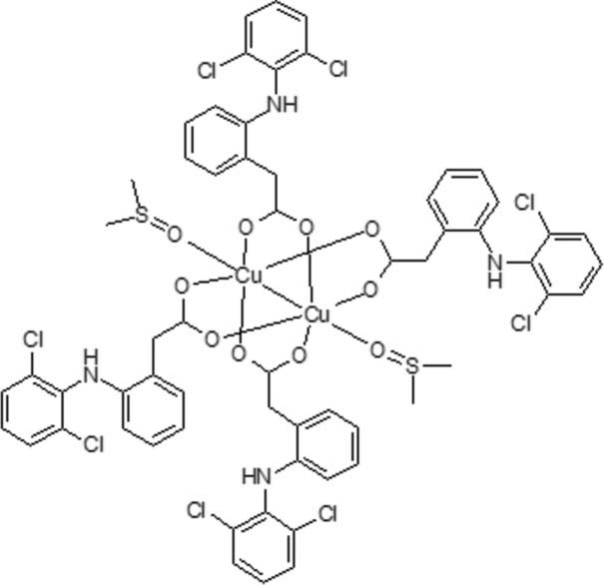


## Experimental

### 

#### Crystal data

[Cu_2_(C_14_H_10_Cl_2_NO_2_)_4_(C_2_H_6_OS)_2_]
*M* *_r_* = 1463.90
Triclinic, 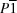

*a* = 10.357 (5) Å
*b* = 12.787 (5) Å
*c* = 12.925 (5) Å
α = 81.605 (5)°
β = 75.561 (5)°
γ = 68.489 (5)°
*V* = 1539.4 (11) Å^3^
*Z* = 1
Mo *K*α radiation
μ = 1.17 mm^−1^
*T* = 100 K
0.30 × 0.21 × 0.18 mm

#### Data collection

Oxford Diffraction SuperNova Atlas diffractometer
Absorption correction: multi-scan (*ABSPACK*; Oxford Diffraction, 2010 ▶) *T*_min_ = 0.867, *T*_max_ = 1.000
42084 measured reflections
10796 independent reflections
9113 reflections with *I* > 2σ(*I*)
*R*_int_ = 0.030

#### Refinement

*R*[*F* ^2^ > 2σ(*F* ^2^)] = 0.031
*wR*(*F* ^2^) = 0.079
*S* = 0.99
10796 reflections
396 parameters
1 restraint
H atoms treated by a mixture of independent and constrained refinement
Δρ_max_ = 0.71 e Å^−3^
Δρ_min_ = −0.51 e Å^−3^

### 

Data collection: *CrysAlis PRO* (Oxford Diffraction, 2010 ▶); cell refinement: *CrysAlis PRO*; data reduction: *CrysAlis PRO*; program(s) used to solve structure: *SHELXS97* (Sheldrick, 2008 ▶); program(s) used to refine structure: *SHELXL97* (Sheldrick, 2008 ▶); molecular graphics: *SHELXTL* (Sheldrick, 2008 ▶); software used to prepare material for publication: *SHELXL97*.

## Supplementary Material

Crystal structure: contains datablock(s) I, global. DOI: 10.1107/S160053681201152X/gg2076sup1.cif

Structure factors: contains datablock(s) I. DOI: 10.1107/S160053681201152X/gg2076Isup2.hkl

Additional supplementary materials:  crystallographic information; 3D view; checkCIF report

## Figures and Tables

**Table:** 

Cu1—O2	1.9647 (11)
Cu1—O1	1.9655 (11)
Cu1—O2′	1.9725 (11)
Cu1—O1′	1.9799 (11)
Cu1—O1*D*	2.1344 (14)
Cu1—Cu1^i^	2.6619 (12)

**Table:** 

O2—Cu1—O1	86.92 (5)
O2—Cu1—O2′	167.83 (4)
O1—Cu1—O2′	92.47 (6)
O2—Cu1—O1′	90.59 (5)
O1—Cu1—O1′	167.60 (4)
O2′—Cu1—O1′	87.41 (6)
O2—Cu1—O1*D*	97.11 (5)
O1—Cu1—O1*D*	94.31 (4)
O2′—Cu1—O1*D*	95.06 (4)
O1′—Cu1—O1*D*	98.06 (4)
O2—Cu1—Cu1^i^	86.45 (4)
O1—Cu1—Cu1^i^	85.35 (3)
O2′—Cu1—Cu1^i^	81.39 (4)
O1′—Cu1—Cu1^i^	82.36 (3)
O1*D*—Cu1—Cu1^i^	176.41 (3)

Symmetry code: (i) 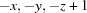.

**Fig. 1.**
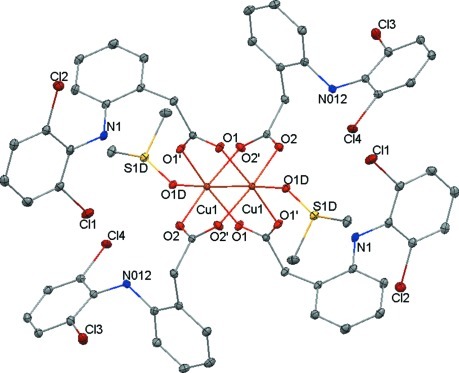
: A representation of the title compound (I) with displacement ellipsoids at the 30% probability level.

**Fig. 2.**
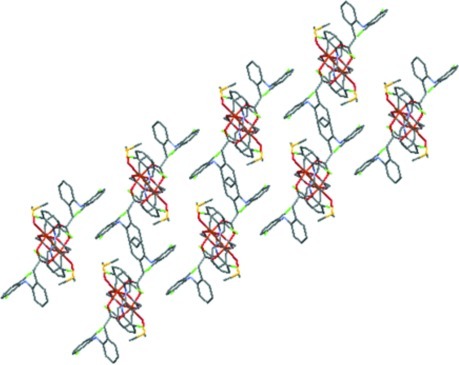
: The π···π stacking interactions in the [Cu2(diclofenac)4(DMSO)2] complex (H atoms are omitted for clarity).

## supplementary crystallographic information

### Comment

The proposed curative properties of Cu-based non-steroidal anti-inflammatory drugs (NSAIDs) have led to the development of numerous Cu(II) complexes of NSAIDs with enhanced anti-inflammatory activity and reduced gastrointestinal toxicity compared with their uncomplexed parent drug (Weder *et al.*, 2002). Furthermore, little is known of their pharmacokinetic and biodistribution profile in both humans and animals, stabilty in biological media, or of the relative potency/efficacy of the Cu^II^ monomeric *versus* Cu^II^ dimeric complexes. The structure of the Cu-NSAID is likely to be an important factor for its biological activity. For example, the anti-tumor activity of the monomeric Cu^II^ complex of aspirin ([Cu(Asp)_2_(py)_2_]) is reportedly more effective than the dimeric [Cu_2_(Asp)_4_] complex (Oberley & Buettner, 1979). Thus, it appears to be essential to obtain structural information on Cu^(II)^ complexes of NSAIDs in order to fully understand their biological activity. Being able to act as a ligand through its carboxylate function of the aromatic ring, different diclofenac complexes (Cu-NSAID complex) were described in the literature. It gives rise to a mononuclear [Cu(diclofenac)_2_(H_2_O)_2_].2H_2_O complex (Sayen *et al.*, 2012) and a binuclear [Cu_2_(diclofenac)_4_(DMF)_2_] complex without a metal-metal bond (Kovala-Demertzi *et al.*, 1997). The former resulted in a distorted octahedral geometry, whereas the latter resulted in a binuclear copper complex where each metal centre is described as a perfect square bipyramid with a DMF oxygen occupying apical position. In order to favour the metal···metal bond, which stabilizes the complex and thus impact the biological activity, we have tried various coordinating solvents during the recrystallization.

The structure of the binuclear [bis(2-[2-(2,6-dichlorophenyl)aminophenyl]ethanoate)bis(DMSO)copper(II)] complex (I) has been obtained. It consists of a quadruply bridged neutral molecule lying on a crystallographic centre of inversion (Fig. 1). Indeed, the four carboxylato moieties act as bridging ligands exhibiting a centre of symmetry midway between the two Cu atoms. The solvent used in the synthesis binds in the position *trans* to the Cu—Cu axis. The dimeric structure has a Cu—Cu distance of 2.6619 (12) Å, with an octahedral stereochemistry tetragonally elongated along the Cu—Cu-O_solvent_ axis due to the Jahn-Teller effect (Table 1).

In the binuclear unit, the carboxylic acids are fully deprotonated to balance the charge from the Cu^II^ ions. The stability of the structure is ensured *via* a network of /p···/p interactions involving the phenyl acetate rings of the diclofenac molecules. On the other hand, no intermolecular H-bonding is observed (Fig. 2).

The use of DMSO solvent allowed the formation of a binuclear complex with a Cu_2_ metal core, which stabilizes the complex in biological media. It was shown that binuclear Cu-NSAID complexes exhibit similar biological activity as the mononuclear complex, but with a higher stability (Dimiza *et al.*, 2011), making them relevant compounds in the treatment of tumor cell lines (Theodorou *et al.*, 1999).

### Experimental

The [bis(2-[2-(2,6-dichlorophenyl)aminophenyl]ethanoate)bis(DMSO)copper(II)] was prepared from a mixture of copper sulfate and diclofenac sodium salt in the molar ratio 1:2 in deionized water. After stirring for 2 hrs at room temperature, the reaction mixture was filtered and the green precipitate was washed with water and dried in air. Crystals suitable for X-ray diffraction measurements were obtained by slow evaporation of a DMSO solution of the complex.

### Crystal data

**Table:**

[Cu_2_(C_14_H_10_Cl_2_NO_2_)_4_(C_2_H_6_OS)_2_]	*Z* = 1
*M**_r_* = 1463.90	*F*(000) = 746
Triclinic, *P*1	*D*_x_ = 1.579 Mg m^−^^3^
Hall symbol: -P 1	Mo *K*α radiation, λ = 0.71073 Å
*a* = 10.357 (5) Å	Cell parameters from 19895 reflections
*b* = 12.787 (5) Å	θ = 3.0–33.3°
*c* = 12.925 (5) Å	µ = 1.17 mm^−^^1^
α = 81.605 (5)°	*T* = 100 K
β = 75.561 (5)°	Prismatic, green
γ = 68.489 (5)°	0.30 × 0.21 × 0.18 mm
*V* = 1539.4 (11) Å^3^	

### Data collection

**Table:**

Oxford Diffraction SuperNova Atlas diffractometer	10796 independent reflections
Radiation source: SuperNova (Mo) X-ray Source	9113 reflections with *I* > 2σ(*I*)
Mirror monochromator	*R*_int_ = 0.030
Detector resolution: 10.4508 pixels mm^-1^	θ_max_ = 33.4°, θ_min_ = 3.0°
CCD scans	*h* = −15→15
Absorption correction: multi-scan (ABSPACK; Oxford Diffraction, 2010)	*k* = −18→19
*T*_min_ = 0.867, *T*_max_ = 1.000	*l* = −19→19
42084 measured reflections	

### Refinement

**Table:**

Refinement on *F*^2^	Primary atom site location: structure-invariant direct methods
Least-squares matrix: full	Secondary atom site location: difference Fourier map
*R*[*F*^2^ > 2σ(*F*^2^)] = 0.031	Hydrogen site location: inferred from neighbouring sites
*wR*(*F*^2^) = 0.079	H atoms treated by a mixture of independent and constrained refinement
*S* = 0.99	*w* = 1/[σ^2^(*F*_o_^2^) + (0.0335*P*)^2^ + 0.9652*P*] where *P* = (*F*_o_^2^ + 2*F*_c_^2^)/3
10796 reflections	(Δ/σ)_max_ = 0.012
396 parameters	Δρ_max_ = 0.71 e Å^−^^3^
1 restraint	Δρ_min_ = −0.51 e Å^−^^3^

### Special details

**Table:**

Geometry. All e.s.d.'s (except the e.s.d. in the dihedral angle between two l.s. planes) are estimated using the full covariance matrix. The cell e.s.d.'s are taken into account individually in the estimation of e.s.d.'s in distances, angles and torsion angles; correlations between e.s.d.'s in cell parameters are only used when they are defined by crystal symmetry. An approximate (isotropic) treatment of cell e.s.d.'s is used for estimating e.s.d.'s involving l.s. planes.
Refinement. Refinement of *F*^2^ against ALL reflections. The weighted *R*-factor *wR* and goodness of fit *S* are based on *F*^2^, conventional *R*-factors *R* are based on *F*, with *F* set to zero for negative *F*^2^. The threshold expression of *F*^2^ > σ(*F*^2^) is used only for calculating *R*-factors(gt) *etc*. and is not relevant to the choice of reflections for refinement. *R*-factors based on *F*^2^ are statistically about twice as large as those based on *F*, and *R*- factors based on ALL data will be even larger.

### Fractional atomic coordinates and isotropic or equivalent isotropic displacement parameters (Å^2^)

**Table:**

	*x*	*y*	*z*	*U*_iso_*/*U*_eq_	
Cu1	0.120933 (16)	0.021416 (13)	0.473791 (12)	0.01201 (4)	
Cl3	−0.08018 (4)	0.53916 (3)	0.12807 (3)	0.02570 (8)	
Cl2	−0.24985 (4)	0.22114 (3)	1.09549 (3)	0.02408 (7)	
S1D	0.46463 (3)	−0.01303 (3)	0.37338 (3)	0.01652 (7)	
Cl4	0.36720 (4)	0.26927 (3)	0.29584 (3)	0.02399 (8)	
Cl1	−0.13597 (4)	−0.16032 (3)	0.90149 (3)	0.02907 (9)	
O2	0.02500 (10)	0.14887 (8)	0.38286 (8)	0.01807 (19)	
O1D	0.31958 (10)	0.04789 (8)	0.43929 (8)	0.01638 (18)	
O1	0.03591 (10)	0.12257 (9)	0.59225 (8)	0.0190 (2)	
O1'	0.16521 (11)	−0.08291 (9)	0.36053 (8)	0.0197 (2)	
O2'	0.17899 (11)	−0.11273 (9)	0.57158 (9)	0.0205 (2)	
N012	0.05900 (13)	0.35894 (10)	0.27795 (10)	0.0178 (2)	
H012	0.097 (2)	0.2948 (17)	0.2976 (15)	0.021*	
C14	−0.21991 (14)	0.07801 (12)	1.10092 (11)	0.0177 (2)	
C28	−0.06437 (14)	0.42098 (11)	0.35062 (11)	0.0169 (2)	
C8	−0.31758 (14)	0.17788 (12)	0.88170 (10)	0.0159 (2)	
N1	−0.19748 (13)	0.08795 (11)	0.90651 (10)	0.0205 (2)	
H1	−0.153 (2)	0.0471 (17)	0.8576 (16)	0.025*	
C31	0.17566 (15)	0.56327 (12)	0.07734 (12)	0.0199 (3)	
H31	0.1352	0.6248	0.0308	0.024*	
C13	−0.21504 (15)	0.01805 (13)	1.19914 (11)	0.0199 (3)	
H13	−0.2323	0.0557	1.2620	0.024*	
C27	−0.07237 (16)	0.51895 (12)	0.39113 (12)	0.0205 (3)	
H27	0.0052	0.5456	0.3691	0.025*	
C2	−0.15089 (14)	0.23216 (12)	0.72217 (11)	0.0162 (2)	
H2A	−0.0860	0.2187	0.7713	0.019*	
H2B	−0.1537	0.3038	0.6800	0.019*	
C9	−0.19799 (14)	0.02709 (12)	1.00581 (11)	0.0176 (3)	
C1	−0.08847 (14)	0.13770 (11)	0.64569 (10)	0.0136 (2)	
C30	0.09412 (15)	0.50313 (12)	0.14053 (11)	0.0187 (3)	
C1D	0.44047 (16)	−0.01951 (14)	0.24247 (11)	0.0219 (3)	
H02A	0.3665	−0.0520	0.2481	0.033*	
H02B	0.5298	−0.0667	0.1995	0.033*	
H02C	0.4118	0.0566	0.2080	0.033*	
C5	−0.55113 (15)	0.35002 (13)	0.82325 (12)	0.0232 (3)	
H5	−0.6306	0.4077	0.8023	0.028*	
C12	−0.18466 (15)	−0.09755 (14)	1.20482 (12)	0.0221 (3)	
H12	−0.1810	−0.1394	1.2718	0.026*	
C11	−0.15969 (15)	−0.15203 (13)	1.11267 (13)	0.0223 (3)	
H11	−0.1375	−0.2313	1.1161	0.027*	
C7	−0.45321 (15)	0.19793 (13)	0.94636 (11)	0.0190 (3)	
H7	−0.4663	0.1523	1.0103	0.023*	
C6	−0.56910 (15)	0.28436 (13)	0.91755 (12)	0.0216 (3)	
H6	−0.6609	0.2986	0.9625	0.026*	
C2D	0.50562 (18)	−0.15937 (13)	0.41473 (14)	0.0281 (3)	
H03A	0.5213	−0.1717	0.4879	0.042*	
H03B	0.5917	−0.2037	0.3665	0.042*	
H03C	0.4263	−0.1828	0.4124	0.042*	
C4	−0.41565 (15)	0.33051 (12)	0.75986 (12)	0.0198 (3)	
H4	−0.4034	0.3757	0.6955	0.024*	
C26	−0.19259 (16)	0.57789 (13)	0.46336 (12)	0.0227 (3)	
H26	−0.1974	0.6449	0.4900	0.027*	
C22	−0.17896 (14)	0.28408 (11)	0.32842 (11)	0.0162 (2)	
H22A	−0.1299	0.2857	0.2522	0.019*	
H22B	−0.2780	0.2907	0.3311	0.019*	
C32	0.31703 (15)	0.53252 (12)	0.08282 (12)	0.0205 (3)	
H32	0.3734	0.5742	0.0412	0.025*	
C10	−0.16740 (15)	−0.09000 (13)	1.01577 (11)	0.0196 (3)	
C21	−0.10505 (14)	0.17246 (11)	0.38384 (10)	0.0142 (2)	
C23	−0.17988 (14)	0.38320 (11)	0.37996 (11)	0.0159 (2)	
C3	−0.29740 (14)	0.24632 (11)	0.78848 (10)	0.0151 (2)	
C24	−0.29898 (15)	0.44231 (12)	0.45452 (12)	0.0201 (3)	
H24	−0.3769	0.4160	0.4769	0.024*	
C25	−0.30575 (16)	0.53873 (13)	0.49652 (13)	0.0243 (3)	
H25	−0.3872	0.5775	0.5475	0.029*	
C33	0.37580 (15)	0.44104 (12)	0.14897 (12)	0.0196 (3)	
H33	0.4735	0.4179	0.1507	0.024*	
C34	0.29167 (14)	0.38323 (11)	0.21269 (11)	0.0167 (2)	
C29	0.14713 (14)	0.41375 (11)	0.21268 (11)	0.0163 (2)	

### Atomic displacement parameters (Å^2^)

**Table:**

	*U*^11^	*U*^22^	*U*^33^	*U*^12^	*U*^13^	*U*^23^
Cu1	0.01134 (7)	0.01206 (8)	0.01214 (7)	−0.00424 (6)	−0.00065 (5)	−0.00190 (5)
Cl3	0.01649 (15)	0.03093 (19)	0.02816 (18)	−0.00870 (14)	−0.00621 (13)	0.00686 (14)
Cl2	0.02819 (18)	0.02315 (17)	0.02436 (16)	−0.01136 (14)	−0.00732 (14)	−0.00237 (13)
S1D	0.01188 (14)	0.01976 (16)	0.01874 (15)	−0.00561 (12)	−0.00296 (11)	−0.00384 (12)
Cl4	0.02377 (17)	0.01794 (16)	0.02430 (16)	−0.00113 (13)	−0.00500 (13)	0.00066 (12)
Cl1	0.02692 (18)	0.02932 (19)	0.02732 (18)	−0.00156 (15)	−0.00549 (14)	−0.01270 (15)
O2	0.0154 (4)	0.0174 (5)	0.0202 (5)	−0.0061 (4)	−0.0037 (4)	0.0033 (4)
O1D	0.0130 (4)	0.0179 (5)	0.0176 (4)	−0.0055 (4)	−0.0002 (3)	−0.0039 (4)
O1	0.0154 (4)	0.0255 (5)	0.0171 (4)	−0.0091 (4)	0.0031 (4)	−0.0097 (4)
O1'	0.0168 (5)	0.0198 (5)	0.0232 (5)	−0.0089 (4)	0.0040 (4)	−0.0112 (4)
O2'	0.0176 (5)	0.0186 (5)	0.0256 (5)	−0.0086 (4)	−0.0057 (4)	0.0057 (4)
N012	0.0174 (5)	0.0128 (5)	0.0200 (5)	−0.0058 (4)	0.0018 (4)	0.0003 (4)
C14	0.0140 (6)	0.0215 (7)	0.0176 (6)	−0.0060 (5)	−0.0030 (5)	−0.0018 (5)
C28	0.0164 (6)	0.0154 (6)	0.0163 (6)	−0.0046 (5)	−0.0010 (5)	−0.0003 (5)
C8	0.0138 (6)	0.0196 (6)	0.0139 (5)	−0.0041 (5)	−0.0031 (4)	−0.0048 (5)
N1	0.0152 (5)	0.0249 (6)	0.0139 (5)	−0.0001 (5)	0.0005 (4)	−0.0023 (4)
C31	0.0186 (6)	0.0176 (6)	0.0207 (6)	−0.0060 (5)	−0.0012 (5)	0.0019 (5)
C13	0.0152 (6)	0.0294 (7)	0.0163 (6)	−0.0087 (5)	−0.0040 (5)	−0.0014 (5)
C27	0.0205 (6)	0.0192 (7)	0.0220 (6)	−0.0080 (5)	−0.0023 (5)	−0.0026 (5)
C2	0.0149 (6)	0.0175 (6)	0.0167 (6)	−0.0064 (5)	−0.0002 (5)	−0.0062 (5)
C9	0.0116 (5)	0.0231 (7)	0.0159 (6)	−0.0038 (5)	−0.0016 (5)	−0.0019 (5)
C1	0.0139 (5)	0.0135 (6)	0.0123 (5)	−0.0031 (4)	−0.0028 (4)	−0.0013 (4)
C30	0.0150 (6)	0.0198 (6)	0.0206 (6)	−0.0070 (5)	−0.0021 (5)	0.0000 (5)
C1D	0.0190 (6)	0.0308 (8)	0.0181 (6)	−0.0108 (6)	−0.0015 (5)	−0.0066 (5)
C5	0.0145 (6)	0.0254 (7)	0.0257 (7)	−0.0015 (5)	−0.0044 (5)	−0.0035 (6)
C12	0.0150 (6)	0.0296 (8)	0.0202 (6)	−0.0072 (5)	−0.0059 (5)	0.0051 (6)
C11	0.0152 (6)	0.0213 (7)	0.0279 (7)	−0.0035 (5)	−0.0062 (5)	0.0012 (6)
C7	0.0157 (6)	0.0244 (7)	0.0157 (6)	−0.0066 (5)	−0.0005 (5)	−0.0033 (5)
C6	0.0124 (6)	0.0288 (8)	0.0220 (6)	−0.0053 (5)	−0.0005 (5)	−0.0074 (6)
C2D	0.0259 (8)	0.0193 (7)	0.0342 (8)	−0.0007 (6)	−0.0085 (6)	−0.0017 (6)
C4	0.0181 (6)	0.0197 (6)	0.0196 (6)	−0.0043 (5)	−0.0032 (5)	−0.0025 (5)
C26	0.0226 (7)	0.0181 (7)	0.0260 (7)	−0.0040 (5)	−0.0040 (6)	−0.0069 (5)
C22	0.0162 (6)	0.0161 (6)	0.0163 (6)	−0.0055 (5)	−0.0050 (5)	0.0013 (5)
C32	0.0190 (6)	0.0197 (7)	0.0217 (6)	−0.0091 (5)	0.0017 (5)	−0.0015 (5)
C10	0.0141 (6)	0.0226 (7)	0.0196 (6)	−0.0023 (5)	−0.0032 (5)	−0.0052 (5)
C21	0.0156 (6)	0.0143 (6)	0.0116 (5)	−0.0044 (5)	−0.0015 (4)	−0.0024 (4)
C23	0.0157 (6)	0.0142 (6)	0.0168 (6)	−0.0041 (5)	−0.0037 (5)	0.0002 (4)
C3	0.0132 (5)	0.0170 (6)	0.0153 (5)	−0.0049 (5)	−0.0006 (4)	−0.0068 (4)
C24	0.0148 (6)	0.0210 (7)	0.0220 (6)	−0.0043 (5)	−0.0015 (5)	−0.0022 (5)
C25	0.0183 (7)	0.0240 (7)	0.0259 (7)	−0.0025 (5)	0.0000 (5)	−0.0084 (6)
C33	0.0147 (6)	0.0199 (7)	0.0233 (6)	−0.0056 (5)	−0.0004 (5)	−0.0056 (5)
C34	0.0168 (6)	0.0135 (6)	0.0177 (6)	−0.0033 (5)	−0.0025 (5)	−0.0020 (5)
C29	0.0164 (6)	0.0140 (6)	0.0175 (6)	−0.0062 (5)	0.0005 (5)	−0.0024 (5)

### Geometric parameters (Å, º)

**Table:**

Cu1—O2	1.9647 (11)	C2—H2B	0.9900
Cu1—O1	1.9655 (11)	C9—C10	1.405 (2)
Cu1—O2'	1.9725 (11)	C1—O1'^i^	1.2592 (17)
Cu1—O1'	1.9799 (11)	C30—C29	1.400 (2)
Cu1—O1D	2.1344 (14)	C1D—H02A	0.9800
Cu1—Cu1^i^	2.6619 (12)	C1D—H02B	0.9800
Cl3—C30	1.7350 (17)	C1D—H02C	0.9800
Cl2—C14	1.7344 (17)	C5—C6	1.389 (2)
S1D—O1D	1.5122 (11)	C5—C4	1.390 (2)
S1D—C1D	1.7889 (16)	C5—H5	0.9500
S1D—C2D	1.7905 (18)	C12—C11	1.388 (2)
Cl4—C34	1.7368 (15)	C12—H12	0.9500
Cl1—C10	1.7406 (16)	C11—C10	1.385 (2)
O2—C21	1.2649 (17)	C11—H11	0.9500
O1—C1	1.2595 (16)	C7—C6	1.389 (2)
O1'—C1^i^	1.2592 (17)	C7—H7	0.9500
O2'—C21^i^	1.2578 (16)	C6—H6	0.9500
N012—C29	1.3959 (18)	C2D—H03A	0.9800
N012—C28	1.4212 (18)	C2D—H03B	0.9800
N012—H012	0.80 (2)	C2D—H03C	0.9800
C14—C13	1.386 (2)	C4—C3	1.392 (2)
C14—C9	1.405 (2)	C4—H4	0.9500
C28—C27	1.394 (2)	C26—C25	1.388 (2)
C28—C23	1.397 (2)	C26—H26	0.9500
C8—C7	1.3966 (19)	C22—C23	1.511 (2)
C8—C3	1.401 (2)	C22—C21	1.5198 (19)
C8—N1	1.4193 (18)	C22—H22A	0.9900
N1—C9	1.4003 (19)	C22—H22B	0.9900
N1—H1	0.81 (2)	C32—C33	1.384 (2)
C31—C30	1.386 (2)	C32—H32	0.9500
C31—C32	1.387 (2)	C21—O2'^i^	1.2579 (16)
C31—H31	0.9500	C23—C24	1.3992 (19)
C13—C12	1.389 (2)	C24—C25	1.390 (2)
C13—H13	0.9500	C24—H24	0.9500
C27—C26	1.388 (2)	C25—H25	0.9500
C27—H27	0.9500	C33—C34	1.386 (2)
C2—C3	1.5050 (19)	C33—H33	0.9500
C2—C1	1.5203 (19)	C34—C29	1.402 (2)
C2—H2A	0.9900		
			
O2—Cu1—O1	86.92 (5)	H02A—C1D—H02C	109.5
O2—Cu1—O2'	167.83 (4)	H02B—C1D—H02C	109.5
O1—Cu1—O2'	92.47 (6)	C6—C5—C4	119.31 (14)
O2—Cu1—O1'	90.59 (5)	C6—C5—H5	120.3
O1—Cu1—O1'	167.60 (4)	C4—C5—H5	120.3
O2'—Cu1—O1'	87.41 (6)	C11—C12—C13	120.00 (14)
O2—Cu1—O1D	97.11 (5)	C11—C12—H12	120.0
O1—Cu1—O1D	94.31 (4)	C13—C12—H12	120.0
O2'—Cu1—O1D	95.06 (4)	C10—C11—C12	119.50 (15)
O1'—Cu1—O1D	98.06 (4)	C10—C11—H11	120.3
O2—Cu1—Cu1^i^	86.45 (4)	C12—C11—H11	120.3
O1—Cu1—Cu1^i^	85.35 (3)	C6—C7—C8	120.25 (13)
O2'—Cu1—Cu1^i^	81.39 (4)	C6—C7—H7	119.9
O1'—Cu1—Cu1^i^	82.36 (3)	C8—C7—H7	119.9
O1D—Cu1—Cu1^i^	176.41 (3)	C5—C6—C7	120.16 (13)
O1D—S1D—C1D	106.71 (7)	C5—C6—H6	119.9
O1D—S1D—C2D	106.52 (7)	C7—C6—H6	119.9
C1D—S1D—C2D	98.13 (8)	S1D—C2D—H03A	109.5
C21—O2—Cu1	120.01 (9)	S1D—C2D—H03B	109.5
S1D—O1D—Cu1	133.22 (6)	H03A—C2D—H03B	109.5
C1—O1—Cu1	121.65 (9)	S1D—C2D—H03C	109.5
C1^i^—O1'—Cu1	124.30 (9)	H03A—C2D—H03C	109.5
C21^i^—O2'—Cu1	125.79 (9)	H03B—C2D—H03C	109.5
C29—N012—C28	119.75 (12)	C5—C4—C3	121.55 (14)
C29—N012—H012	116.5 (14)	C5—C4—H4	119.2
C28—N012—H012	115.4 (14)	C3—C4—H4	119.2
C13—C14—C9	122.73 (14)	C25—C26—C27	119.88 (14)
C13—C14—Cl2	118.27 (11)	C25—C26—H26	120.1
C9—C14—Cl2	118.98 (11)	C27—C26—H26	120.1
C27—C28—C23	120.00 (13)	C23—C22—C21	111.97 (11)
C27—C28—N012	121.21 (13)	C23—C22—H22A	109.2
C23—C28—N012	118.79 (13)	C21—C22—H22A	109.2
C7—C8—C3	120.06 (13)	C23—C22—H22B	109.2
C7—C8—N1	121.86 (13)	C21—C22—H22B	109.2
C3—C8—N1	118.07 (12)	H22A—C22—H22B	107.9
C9—N1—C8	123.36 (12)	C33—C32—C31	119.92 (13)
C9—N1—H1	111.4 (14)	C33—C32—H32	120.0
C8—N1—H1	113.6 (14)	C31—C32—H32	120.0
C30—C31—C32	119.23 (13)	C11—C10—C9	122.69 (14)
C30—C31—H31	120.4	C11—C10—Cl1	118.58 (12)
C32—C31—H31	120.4	C9—C10—Cl1	118.73 (11)
C14—C13—C12	119.40 (14)	O2'^i^—C21—O2	125.72 (13)
C14—C13—H13	120.3	O2'^i^—C21—C22	117.28 (12)
C12—C13—H13	120.3	O2—C21—C22	116.98 (12)
C26—C27—C28	120.68 (14)	C28—C23—C24	118.49 (13)
C26—C27—H27	119.7	C28—C23—C22	120.72 (12)
C28—C27—H27	119.7	C24—C23—C22	120.72 (13)
C3—C2—C1	115.83 (11)	C4—C3—C8	118.60 (12)
C3—C2—H2A	108.3	C4—C3—C2	120.36 (13)
C1—C2—H2A	108.3	C8—C3—C2	121.01 (12)
C3—C2—H2B	108.3	C25—C24—C23	121.42 (14)
C1—C2—H2B	108.3	C25—C24—H24	119.3
H2A—C2—H2B	107.4	C23—C24—H24	119.3
N1—C9—C14	122.05 (14)	C26—C25—C24	119.44 (14)
N1—C9—C10	122.15 (13)	C26—C25—H25	120.3
C14—C9—C10	115.66 (13)	C24—C25—H25	120.3
O1'^i^—C1—O1	125.60 (12)	C32—C33—C34	119.82 (13)
O1'^i^—C1—C2	117.90 (12)	C32—C33—H33	120.1
O1—C1—C2	116.48 (12)	C34—C33—H33	120.1
C31—C30—C29	122.77 (13)	C33—C34—C29	122.24 (13)
C31—C30—Cl3	118.42 (11)	C33—C34—Cl4	119.09 (11)
C29—C30—Cl3	118.81 (10)	C29—C34—Cl4	118.66 (10)
S1D—C1D—H02A	109.5	N012—C29—C30	120.60 (13)
S1D—C1D—H02B	109.5	N012—C29—C34	123.50 (13)
H02A—C1D—H02B	109.5	C30—C29—C34	115.89 (12)
S1D—C1D—H02C	109.5		
			
O1—Cu1—O2—C21	−80.75 (10)	C3—C8—C7—C6	1.2 (2)
O2'—Cu1—O2—C21	6.7 (3)	N1—C8—C7—C6	−177.75 (13)
O1'—Cu1—O2—C21	87.09 (10)	C4—C5—C6—C7	−1.9 (2)
O1D—Cu1—O2—C21	−174.72 (10)	C8—C7—C6—C5	1.2 (2)
Cu1^i^—Cu1—O2—C21	4.78 (10)	C6—C5—C4—C3	0.2 (2)
C1D—S1D—O1D—Cu1	54.37 (10)	C28—C27—C26—C25	0.5 (2)
C2D—S1D—O1D—Cu1	−49.71 (10)	C30—C31—C32—C33	1.5 (2)
O2—Cu1—O1D—S1D	−106.94 (9)	C12—C11—C10—C9	0.8 (2)
O1—Cu1—O1D—S1D	165.63 (8)	C12—C11—C10—Cl1	−179.88 (11)
O2'—Cu1—O1D—S1D	72.77 (9)	N1—C9—C10—C11	176.25 (13)
O1'—Cu1—O1D—S1D	−15.32 (9)	C14—C9—C10—C11	0.3 (2)
O2—Cu1—O1—C1	84.24 (11)	N1—C9—C10—Cl1	−3.02 (18)
O2'—Cu1—O1—C1	−83.59 (11)	C14—C9—C10—Cl1	−179.00 (10)
O1'—Cu1—O1—C1	5.6 (3)	Cu1—O2—C21—O2'^i^	−9.74 (19)
O1D—Cu1—O1—C1	−178.85 (10)	Cu1—O2—C21—C22	168.74 (9)
Cu1^i^—Cu1—O1—C1	−2.44 (10)	C23—C22—C21—O2'^i^	116.54 (13)
O2—Cu1—O1'—C1^i^	−93.06 (12)	C23—C22—C21—O2	−62.08 (16)
O1—Cu1—O1'—C1^i^	−14.8 (3)	C27—C28—C23—C24	−3.4 (2)
O2'—Cu1—O1'—C1^i^	74.93 (11)	N012—C28—C23—C24	177.69 (13)
O1D—Cu1—O1'—C1^i^	169.68 (11)	C27—C28—C23—C22	173.79 (13)
Cu1^i^—Cu1—O1'—C1^i^	−6.72 (11)	N012—C28—C23—C22	−5.15 (19)
O2—Cu1—O2'—C21^i^	2.2 (3)	C21—C22—C23—C28	85.23 (16)
O1—Cu1—O2'—C21^i^	89.04 (12)	C21—C22—C23—C24	−97.69 (15)
O1'—Cu1—O2'—C21^i^	−78.54 (11)	C5—C4—C3—C8	2.1 (2)
O1D—Cu1—O2'—C21^i^	−176.41 (11)	C5—C4—C3—C2	−175.91 (13)
Cu1^i^—Cu1—O2'—C21^i^	4.12 (11)	C7—C8—C3—C4	−2.8 (2)
C29—N012—C28—C27	−26.4 (2)	N1—C8—C3—C4	176.17 (12)
C29—N012—C28—C23	152.56 (13)	C7—C8—C3—C2	175.18 (12)
C7—C8—N1—C9	−13.2 (2)	N1—C8—C3—C2	−5.80 (19)
C3—C8—N1—C9	167.83 (13)	C1—C2—C3—C4	−100.88 (15)
C9—C14—C13—C12	1.3 (2)	C1—C2—C3—C8	81.12 (16)
Cl2—C14—C13—C12	−177.01 (11)	C28—C23—C24—C25	2.1 (2)
C23—C28—C27—C26	2.1 (2)	C22—C23—C24—C25	−175.05 (13)
N012—C28—C27—C26	−178.99 (14)	C27—C26—C25—C24	−1.8 (2)
C8—N1—C9—C14	−63.1 (2)	C23—C24—C25—C26	0.5 (2)
C8—N1—C9—C10	121.19 (16)	C31—C32—C33—C34	−2.6 (2)
C13—C14—C9—N1	−177.33 (13)	C32—C33—C34—C29	0.3 (2)
Cl2—C14—C9—N1	0.95 (18)	C32—C33—C34—Cl4	−179.55 (11)
C13—C14—C9—C10	−1.3 (2)	C28—N012—C29—C30	−59.39 (19)
Cl2—C14—C9—C10	176.94 (10)	C28—N012—C29—C34	121.85 (15)
Cu1—O1—C1—O1'^i^	8.65 (19)	C31—C30—C29—N012	177.19 (14)
Cu1—O1—C1—C2	−169.98 (9)	Cl3—C30—C29—N012	−3.74 (19)
C3—C2—C1—O1'^i^	3.49 (18)	C31—C30—C29—C34	−4.0 (2)
C3—C2—C1—O1	−177.76 (12)	Cl3—C30—C29—C34	175.12 (10)
C32—C31—C30—C29	1.9 (2)	C33—C34—C29—N012	−178.30 (13)
C32—C31—C30—Cl3	−177.23 (11)	Cl4—C34—C29—N012	1.52 (19)
C14—C13—C12—C11	−0.1 (2)	C33—C34—C29—C30	2.9 (2)
C13—C12—C11—C10	−0.9 (2)	Cl4—C34—C29—C30	−177.30 (10)

Symmetry code: (i) −*x*, −*y*, −*z*+1.
